# Supplementary material for: Reconciling Mining with the Conservation of Cave Biodiversity: A Quantitative Baseline to Help Establish Conservation Priorities
Source: PLoS One. 2016 Dec 20;11(12):e0168348. doi: 10.1371/journal.pone.0168348 (PMC5173368; doi:10.1371/journal.pone.0168348)
Supplement: S1 Dataset — (ZIP) [file pone.0168348.s002.zip › Taxa/Serra Norte/SN_2013/N4WS/Lista N4WS-03.pdf]

CAVIDADE N4WS-0003

Classe	Ordem	Fam/Outros	Gên/Outros	Espécie	S-U	U-S	Zon.
Annelida	Oligochaeta			sp.		1	P
Arachnida	Acari	(Parasitengonina)		sp.1		X	P
Arachnida	Acari	Macronyssidae		sp.1		X	E
Arachnida	Amblypygi	Phrynidae	*Heterophrynus*	*longicornis*	2	1	P
Arachnida	Araneae	Drymusidae		jovem	X		E
Arachnida	Araneae	Ochyroceratidae		jovem	X	X	E/P
Arachnida	Araneae	Pholcidae	Ninetinae	sp.1		X	E
Arachnida	Araneae	Pisauridae		jovem	1		E
Arachnida	Araneae	Scytodidae	*Scytodes*	*globula*	5	7	E/P
Arachnida	Araneae	Theridiosomatidae	*Plato*	sp.1	X	X	E/P
Arachnida	Opiliones	Sclerosomatidae		jovem	X	X	E
Arachnida	Schizomida	Hubbardiidae	aff. *Rowlandius*	jovem	X		E/P
Chilopoda	Scutigeromorpha	Pselliodidae		jovem	1		E
Diplopoda	Polydesmida	Pyrgodesmidae		sp.1	X	X	E/P
Entognatha	Collembola	Paronellidae		sp.4	X	X	E/P
Insecta	Coleoptera	Staphylinidae	Pselaphinae	sp.2		X	P
Insecta	Coleoptera	Staphylinidae		sp.35		X	E/P
Insecta	Diptera	Ceratopogonidae		jovem		X	E
Insecta	Diptera	Chaoboridae		sp.	X		P
Insecta	Diptera	Phoridae	Metopininae	sp.		X	P
Insecta	Diptera	Psychodidae	*Pintomyia*	*gruta*	X	X	E/P
Insecta	Diptera	Tipulidae	Tipulinae	sp.	X	X	E/P
Insecta	Heteroptera	Cydnidae	Cydninae	jovem	X		E
Insecta	Heteroptera	Veliidae	*Paravelia*	sp.1	X	X	E/P
Insecta	Hymenoptera	Diapriidae		sp.1	X		E
Insecta	Hymenoptera	Formicidae	*Gnamptogenys*	sp.2	X		E
Insecta	Hymenoptera	Formicidae	*Nylanderia*	sp.3		X	E
Insecta	Hymenoptera	Formicidae	*Pheidole*	sp.2	X		E
Insecta	Hymenoptera	Formicidae	*Solenopsis*	sp.1	X		E/P
Insecta	Lepidoptera	Noctuoidea		sp.12	1		E
Insecta	Lepidoptera			jovem		X	P
Insecta	Orthoptera	Phalangopsidae	*Phalangopsis*	sp.	20	180	P
Insecta	Psocoptera	Psyllipsocidae		jovem		X	E/P
Insecta	Thysanura	Nicoletiidae		jovem	X		E
Malacostraca	Isopoda	Armadillidae		sp.1		X	E
Malacostraca	Isopoda	Philosciidae		sp.1	X	X	E/P
Malacostraca	Isopoda	Scleropactidae	*Circoniscus*	*buckupi*		X	E
Mammalia	Chiroptera	Phyllostomidae	*Carollia*	*perspicillata*	1		P
